# Association between Oxytocin Receptor Gene Polymorphisms and Self-Rated ‘Empathic Concern’ in Schizophrenia

**DOI:** 10.1371/journal.pone.0051882

**Published:** 2012-12-20

**Authors:** Christiane Montag, Eva-Maria Brockmann, Anja Lehmann, Daniel J. Müller, Dan Rujescu, Jürgen Gallinat

**Affiliations:** 1 Department of Psychiatry and Psychotherapy, Charité Campus Mitte, Charité University Medicine Berlin (Charité Universitätsmedizin Berlin), Berlin, Germany; 2 Neurogenetics Section, Centre for Addiction and Mental Health, Department of Psychiatry, University of Toronto, Toronto, Ontario, Canada; 3 Department of Psychiatry, University of Munich (Ludwig-Maximilians-Universität München), Munich, Germany; 4 Department of Psychiatry, University of Halle-Wittenberg, Halle, Germany; University of Wuerzburg, Germany

## Abstract

The nonapeptide oxytocin (OXT) and its receptor (OXTR) have been implicated in social cognition, empathy, emotion and stress regulation in humans. Previous studies reported associations between OXT and OXTR genetic polymorphisms and risk for disorders characterized by impaired socio-emotional functioning, such as schizophrenia and autism. Here we investigate the influence of two single nucleotide polymorphisms (SNPs) within the OXTR gene on a measure of socio-emotional functioning in schizophrenic patients. OXTR SNPs that were previously investigated in other studies were genotyped in 145 patients diagnosed with schizophrenia according to DSM-IV and 145 healthy controls matched for age and gender. The Interpersonal Reactivity Index (IRI) was used to assess cognitive (‘perspective taking’), affective (‘empathic concern’) and self-related (‘personal distress’) dimensions of empathy. No group differences in genotype frequencies were observed. MANCOVA revealed a significant main (F [1,282] = 10.464; p<0.01) and interaction effect (genotype by diagnosis: F [1,282] = 4.329; p<0.05) of OXTR SNP rs2254298(A>GG) with ‘empathic concern’. Within the schizophrenia group, linear regression analysis determined OXTR rs2254298 genotype, PANSS negative and general symptom score, and age of disease onset as being significantly associated with ‘empathic concern’. OXTR rs2254298 significantly impacted PANSS general psychopathology scores. No associations were found for OXTR rs53576, IRI ‘perspective taking’ or ‘personal distress’ ratings. Our preliminary findings support hypotheses about an involvement of OXTR rs2254298 in emotional empathy in schizophrenic and healthy individuals, warranting independent replication.

## Introduction

Social-cognitive deficits are an important clinical feature of schizophrenia and have drawn much attention over the past decades [1] as disturbances of the ‘social brain’ essentially impact psychosocial proficiency [2], [3] and might represent trait-markers for this disease [4]. While a large body of evidence confirms impairments of theory of mind [5]–[7] and of various aspects of emotion processing [8], [9], research on empathy in the narrow sense has been published less frequently in schizophrenia [10], [11].

Across species, a broad spectrum of social and emotional behaviors is modulated by the neurohormone oxytocin (OXT) [12]–[15]. OXT is produced in the hypothalamic paraventricular (PVN) and supraoptic nuclei (SON). Their magnocellular neurons mainly project to the neurohypophysis, whereas central OXT, after its axonal or somatodendritic release, can modulate functional activity in many brain regions including cortical areas, amygdala, striatum, nucleus accumbens, hippocampus, ventral tegmental area and brainstem nuclei [16]–[21]. Animal studies demonstrate that OXT stimulates not only reproductive behaviors, but also has positive effects on social recognition, affiliation and approach behavior while alleviating social stress and anxiety [12], [13], [18], [20], [22]–[25]. In humans, a substantial body of research indicates its regulative function for social cognition [26]–[28], social memory [29]–[31], prosocial behavior [32]–[34], attachment [35] and trust [36]. Specifically, OXT has been shown to dampen the hypothalamic-pituitary-adrenal-axis (HPA-axis) [37], [38] and to reduce amygdala activation in response to social stressors [39]–[42]. Of note, OXT interacts with the dopaminergic system and thus stimulates the attribution of salience to social and emotional stimuli [43]–[46].

Alterations of the central oxytocinergic system might play a role in the pathogenesis of disorders marked by social deficits such as schizophrenia or modulate their presentation [47]. This assumption is supported by preliminary evidence about beneficial effects of high plasma OXT levels [48]–[50] or intranasal OXT administration on psychotic symptoms [51]–[53] and also on theory of mind, social perception [52], [54] or verbal memory [55] in schizophrenia. Evidence from animal studies suggests a role of OXT as a mediator of second-generation antipsychotic action [56], [57], and its ability to restore glutamatergic dysfunction induced by NMDA-receptor-antagonists [58], [59].

Beside peripheral OXT concentrations, variations of the OXTR gene might contribute to explain variability of core socio-emotional processes and related phenotypes. OXTR has been discussed as a candidate gene for autism spectrum vulnerability [60]–[68], but until to date only few studies have investigated variations of oxytocinergic system genes in schizophrenia [69]–[72]. Souza et al. first reported the association of three OXT polymorphisms with the diagnosis of schizophrenia (rs4813625, rs3761248 in a case-control, and nominal over-transmission of rs2740204 in a family-based study) [70]. OXT SNP rs2740204 was shown to be related to clozapine treatment response, while OXTR variants were associated to overall symptoms (rs237885, rs237887) and improvement in positive symptoms (rs11706648, rs4686301, rs237899) [69]. Teltsh et al. determined three SNPs within the OXT-AVP cluster (rs4813626, rs2740204, AVP3011589) that were associated with schizophrenia in a family-based association study [71]. Montag et al. identified OXTR SNP rs53576(A) as being linked to the disease in a case-control study of 406 schizophrenic and 406 healthy individuals; rs53576 was associated with PANSS general psychopathology, and rs237902 with negative symptoms [72].

However, this study sets out to explore potential associations of trait empathy in schizophrenic and healthy individuals with two selected OXTR SNPs - both situated in the third intron of OXTR - that were consistently linked to socio-emotional phenotypes before. Beside the single report of being associated with schizophrenia [72], OXTR rs53576 was suggested to mediate dispositional empathy [73], social stress reactivity [73]–[75], prosocial attitude [76], social support seeking [77] and trust [78], while OXTR rs2254298 was associated with cognitive empathy in healthy individuals [79]. OXTR rs2254298 [80], [81] as well as rs53576 [82], [83] were linked to attachment measures and parental sensitivity. Both OXTR SNPs were also hypothesized to be associated with unipolar depression [82] and with risk for autism spectrum disorder [60], [62], [63], [67], [84]. Moreover, our selection of SNPs was guided by recent evidence from imaging studies indicating an impact of OXTR rs2254298 [85]–[87] and OXTR rs53576 [88] on key oxytocinergic structures including the amygdala and the hypothalamus. Trait empathy was examined using the Interpersonal Reactivity Index [89] which covers three essential dimensions of empathic responding - the cognitive facet of empathy (‘perspective taking’) as well as altruistic concern (‘empathic concern’) and the self-directed, aversive experience of social distress (‘personal distress’). We hypothesized that OXTR risk allele carriers would show deficits in self-rated empathic dimensions.

## Materials and Methods

### Ethics Statement

The study was approved by the local ethics committee (Charité Universitätsmedizin Berlin, Germany). All subjects gave written informed consent. The study was conducted according to the principles expressed in the Declaration of Helsinki. All potential participants who declined to participate or otherwise did not participate were eligible for treatment and were not disadvantaged in any other way by not participating in the study.

Only patients with an unaltered capacity to consent were included in the study. Capacity to consent was confirmed during the screening process by both the treating physician, who was not involved in the study, and C.M. according to the criteria developed by [90].

### Participants

Schizophrenic subjects (n = 145), aged between 18 and 69 years, recruited from the Department of Psychiatry, Charité Universitätsmedizin Berlin, Campus Mitte, participated in the study. Diagnosis was confirmed using the Structured Clinical Interview for DSM-IV (SCID-I; German version) [91]; symptom severity was assessed with the Positive and Negative Syndrome Scale [92]. Healthy control subjects (n = 145) were recruited by newspaper advertisements and screened by trained psychiatrists with a structured interview (M.I.N.I.) [93]. All together, normal controls were matched to the patients’ sample according to age and gender. All participants were of European descent and not related to each other. Exclusion criteria for both groups were DSM-IV axis-I or axis-II disorders (except schizophrenia for the patient group). Controls reporting axis-I mental disorders in their first- or second degree relatives were excluded. The partial overlap of the study sample with the participants of a previous study [72] was limited by the availability of data on empathy, and only a fraction of participants could be retrieved to take part in the current project. Moreover, OXTR SNP rs2254298 had not been genotyped at the time, when [72] was prepared. EDTA blood was taken from all participants for genotyping. Clinical types of schizophrenia according to DSM-IV-TR were as follows: paranoid (n = 128), undifferentiated (n = 5), disorganized (n = 4), catatonic (n = 3), residual (n = 1), and in n = 4 schizoaffective disorder was reported.

### Genotyping

DNA extraction was done with the QIAamp Blood Maxi Kit (QIAamp DNA Blood Midi/Maxi Handbook, Firma Qiagen, Hilden, Germany, 2005). DNA concentration was adjusted using the PicoGreen quantitation reagent (Invitrogen, Karlsruhe, Germany). One ng DNA was genotyped using the iPLEX assay on the MassARRAY MALDI-TOF mass spectrometer (SEQUENOM, Hamburg, Germany). Genotyping call rates in cases and controls were all >99%. Allele frequencies were similar to CEU sample frequencies (www.hapmap.org).

### Interpersonal Reactivity Index

The Interpersonal Reactivity Index (IRI) [89] assesses aspects of empathic responding, which were determined by factor analysis. We used the German translation (‘Saarbrücker Persönlichkeitsfragebogen’; SPF) [94]. For analysis, three relevant IRI-subscales were used: ‘Perspective taking’ refers to the tendency to spontaneously adopt the psychological point of view of others and to reason about their mental states. The ‘empathic concern’ scale comprises respondents’ prosocial feelings of warmth, compassion and concern for others. ‘Personal distress’ measures self-oriented feelings of anxiety and discomfort in response to the distress of others. Construct validity, internal consistency the IRI scales [89] and its feasibility in schizophrenic patients were supported in several studies [3], [11], [95].

### General Cognitive Function

A multiple choice vocabulary test (Mehrfachwahlwortschatztest, MWT-B) [96] was applied to estimate verbal intelligence.

### Statistical Analysis

These were carried out as indicated in the results section using PASW for Windows 20.0® and code for mediation analysis available from http://www.afhayes.com/ [97]. Statistical significance was set at p<0.05. Genotypes were tested to conform to the Hardy-Weinberg-equilibrium using HWSIM Software (http://krunch.med.yale.edu/hwsim/). Allelic and genotypic distributions and odds ratios (OR) between patients and controls were examined by Pearson χ^2^ test on 2×2 contingency tables. Multivariate analysis of variance and linear regression analyses were performed as described in the following section. Because of the low minor allele frequency of OXTR rs2254298 AA genotype (n = 2 in each group) and of OXTR rs53576 AA genotype (schizophrenia: n = 7; controls: n = 13) they were combined with the heterozygotes for statistical analyses in a presumably dominant genetic model.

## Results

Demographic data and disease characteristics of schizophrenic participants are given in **Table 1**. Schizophrenia patients and healthy controls differed significantly in verbal IQ and educational years. All genotype frequencies of rs2254298 and rs53576 were in accordance with the Hardy-Weinberg-equilibrium in the schizophrenic, healthy and combined samples (p>0.05). As for OXTR rs2254298, A-allele frequencies were 0.11 in the schizophrenia and 0.10 in the healthy sample; for OXTR rs53576, A-allele frequencies were 0.32 in the schizophrenia and 0.29 in the healthy sample. Chi-squared tests revealed no significant differences of genotype distributions and allele frequencies for the studied SNPs between groups, and frequencies were also unrelated to gender in the overall sample (χ^2^ test, p>0.05). However, in the patients group, the AA- or AG-genotype of rs2254298 was significantly more common in males than in females (χ^2^ = 5.379, p = 0.020).

**Table 1:** Demographic data and disease characteristics in schizophrenic patients (n = 145) and controls (n = 145); between-group comparisons.

	Schizophrenic patients	Healthy controls	Statistics
**Age (mean years±SD)**	36.9±10.6	37.2±12.0	T = −0.244, p>0.052)
**Gender (m/f)**	91/54	79/66	?^2^ = 0.153, p>0.053)
**Education (mean years±SD)**	13.0±2.9	15.1±2.2	**T = −7.056, p<0.001** 2)
**Verbal IQ (mean years±SD)**	103.9±13.5	108.9±13.4	**T = −3.178, p<0.01** 2)
**Age at first episode [yrs.]**	26.5±8.4	–	–
**Duration of illness [yrs.]**	10.4±9.5	–	–
**Neuroleptic dose** 1)	453.8±373.6	–	–
**PANSS positive score**	17.0±6.4	–	–
**PANSS negative score**	19.4±7.8	–	–
**PANSS general score**	35.6±10.7	–	–

1) dose equivalent to [mg] Chlorpromazine;

2) T-test for independent samples (two-sided);

3) χ^2^-Test.

Significant results are indicated in bold type.

As for the IRI results, schizophrenia patients showed significantly lower scores for ‘perspective taking’ scores and higher scores for ‘personal distress’ than healthy individuals (**Table 2**). T-tests revealed no differences between sexes in either group (t-test for independent samples, p>0.05).

**Table 2:** Self rated dimensions of empathy (Interpersonal Reactivity Index, IRI) in schizophrenic patients (n = 145) and controls (n = 145); between-group comparisons.

	Schizophrenic patients	Healthy controls	Statistics1
**IRI ‘perspective taking’**	23.1±4.7	24.6±3.8	**T = −3.085, p<0.01** 1)
**IRI ‘empathic concern’**	25.3±4.5	25.6±3.6	T = −0.609, p>0.051)
**IRI ‘personal distress’**	20.1±4.5	15.8±4.2	**T = −8.231, p<0.001** 1)

1) T-test for independent samples (two-sided). Significant results are indicated in bold type.

### OXTR Polymorphisms, IRI Dimensions of Empathy and Diagnostic Group

**Figure 1:**
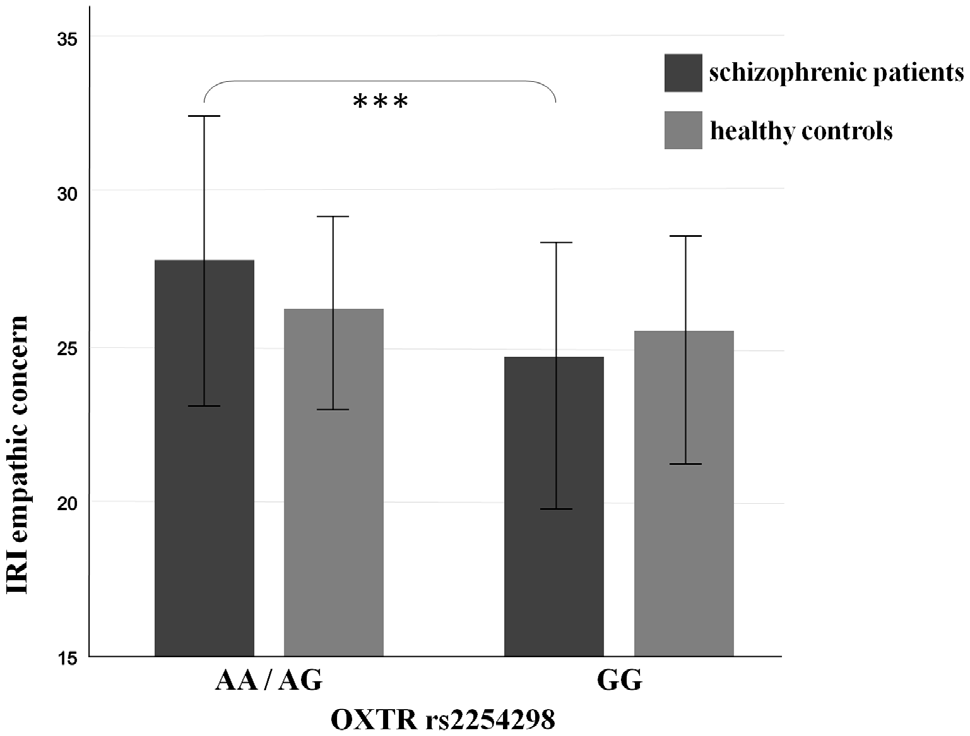
OXTR rs2254298 polymorphisms and IRI ‘empathic concern’ scores in schizophrenic patients and healthy controls. Self-rated IRI ‘empathic concern’ scores are significantly higher in schizophrenic patients endowed with an OXTR SNP rs2254298 AA- or AG-genotype compared to GG-genotype carriers (n = 145; mean, SD; t-test for independent samples, ***: p<0.001), while no significant differences between genotypes are detected in healthy controls (n = 145; p>0.05).

Schizophrenic patients carrying one or two OXTR rs2254298 A-alleles showed significantly more ‘empathic concern’ than those carrying two G-alleles **(** **Table 3** **;** **Figure 1** **).** Multivariate analysis of covariance (MANCOVA) was computed to determine main and interaction effects of rs2254298 and rs53576 genotypes, and diagnosis as factors, as well as the covariate verbal IQ on the three relevant IRI scores as dependent variables (‘perspective taking’, ‘empathic concern’, ‘personal distress’). Homoscedasticity of samples was confirmed by Box-M and Levene tests (p>0.05). Significant overall effects were detected for OXTR rs2254298, diagnostic group, and IQ; the interaction between OXTR rs2254298 genotype and diagnosis was significant (**Table 4**). Post-hoc analyses indicated higher IRI ‘empathic concern’ in the combined OXTR AA/AG-genotype (mean = 27.1, SD = 4.1) compared to GG (mean = 25.1, SD = 4.0). The variable diagnostic group showed significant impact on ‘perspective taking’ (SZ: mean = 23.1, SD = 4.7; HC: mean = 24.6, SD = 3.8) and on ‘personal distress’ (SZ: mean = 20.1, SD = 4.6; HC: mean = 15.8, SD = 4.3). Diagnosis showed no significant main effect on ‘empathic concern’, but schizophrenic patients carrying one or two A-alleles of OXTR rs2254298 showed highest ‘empathic concern’ compared to all the other groups. Verbal IQ was positively associated with IRI ‘perspective taking’. As the introduction of gender as an additional factor would have led to small cell sizes and heterogeneity of covariance matrices, another MANCOVA was performed to determine the influence of gender, OXTR rs2254298 and diagnosis on ‘empathic concern’ scores, while controlling for cognition more strictly (factors: OXTR rs2254298, gender and diagnosis; covariates: verbal IQ and educational years). While females showed significantly higher values of IRI ‘empathic concern’ and ‘personal distress’, the significant impact of OXTR rs2254298 on ‘empathic concern’ remained, and there was no significant interaction between gender and OXTR rs2254298. (**supporting information: Table S1**).

**Table 3:** Raw data of IRI scores (mean, SD) by OXTR rs2254298 and rs53576 genotypes in schizophrenia patients (SZ, n = 145) and healthy controls (HC, n = 145).

OXTR Genotypes	IRI scores					
	‘perspective taking’		‘empathic concern’		‘personal distress’	
	SZ	HC	SZ1)	HC	SZ	HC
**rs2254298(AA/AG)**	23.4±4.2	25.8±4.0	**27.8±4.7**	26.2±3.1	20.5±4.8	14.8±4.6
**rs2254298(GG)**	23.0±4.9	24.4±3.7	**24.7±4.3**	25.5±3.6	20.0±4.5	16.1±4.2
**rs53576(AA/AG)**	23.1±4.7	24.4±3.7	25.3±4.6	25.5±3.4	20.0±4.2	15.6±4.3
**rs53576(GG)**	23.0±4.8	24.9±3.9	25.4±4.6	25.8±3.8	20.2±5.1	16.1±4.2

1) T-test for independent samples: T = −3.493, p<0.001.

**Table 4:** MANCOVA of 3 IRI scores in schizophrenia patients (SZ, n = 145) and healthy controls (HC, n = 145); factors: OXTR rs2254298 (GG vs. A carriers), OXTR rs53576 (GG vs. A carriers) and diagnosis, covariate: verbal IQ.

	OXTR s2254298	OXTR s53576	Diagnosis	OXTR s2254298 x Diagnosis	OXTR rs53576 x Diagnosis	rs225429 x rs53576	Verbal IQ
**MANCOVA F** [3,280] (Effect size)	3.681* (_p_η^2^ = 0.038)	0.536 (_p_η^2^ = 0.006)	20.473***(_p_η^2^ = 0.180)	3.018* (_p_η^2^ = 0.031)	0.199 _p_η^2^ = 0.002)	0.437 _p_η^2^ = 0.005)	3.746*_p_η^2^ = 0.039)
Post hoc ANOVA F [1,282]							
IRI ‘perspective taking’ (R^2^_adj_ = 0.057)	2.388	0.119	6.218*	0.595	0.151	0.208	14.263***
IRI ‘empathic concern’ (R^2^_adj_ = = 0.036)	10.464**	0.042	0.651	4.329*	0.291	0.156	1.548
IRI ‘personal distress’ (R^2^_adj_ = = 0.182)	0.453	1.481	53.697***	2.197	0.280	1.051	0.094

Significant results are indicated in bold type (*p<0.05, **p<0.01, ***p<0.001).

### OXTR Polymorphisms, Disease Characteristics and Empathy in Schizophrenia Patients

**Figure 2:**
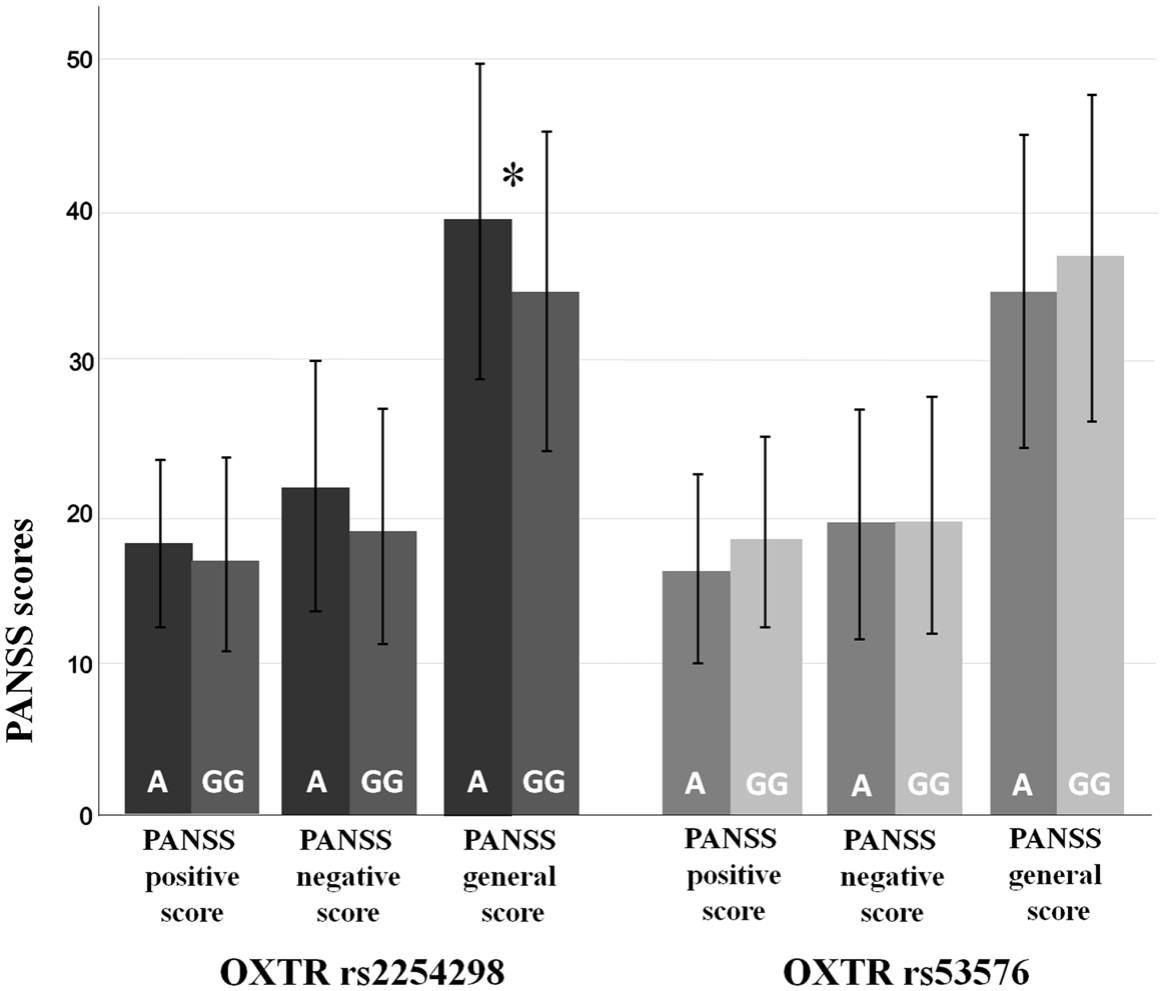
OXTR rs2254298 and rs53576 polymorphisms and PANSS positive, negative and general psychopathology scores in schizophrenic patients. Schizophrenic patients carrying AA- or AG-genotypes of OXTR rs2254298 show significantly higher PANSS general psychopathology scores than GG-carriers (n = 145; mean, SD; t-test for independent samples, *: p<0.05).

Within the patient group, carriers of AA/AG- versus GG-genotypes of OXTR SNP rs2254298 and OXTR SNP rs53576, respectively, did not differ with respect to age, verbal IQ, educational years, age at disease onset, duration of illness, first- and second-generation antipsychotic daily dose and cumulative treatment years with antipsychotics (t-test for independent samples, p>0.05). As for the impact of OXTR SNPs rs2254298 and rs53576 on psychopathological symptom severity, t-tests for independent samples indicated significantly higher values on the PANSS general psychopathology score in patients endowed with one or two A-alleles of rs2254298 (T = −2.355; p = 0.020) (**Figure 2**). No significant group differences were detected for the other PANSS scores and for rs53576.

To explore the association between OXTR polymorphisms and self-rated empathy, linear regression analyses were performed. Dependent variables were the three IRI scores, independent variables were OXTR rs2254289 and rs53576 polymorphisms, gender, age at first manifestation, duration of illness, PANSS positive, negative and general scores. As for IRI ‘empathic concern’, the model predicted 18.1% of total variance (F[8; 136] = 4.979, p<0.001). Significant predictors of ‘empathic concern’ were OXTR rs2254298 genotype (β = −0.307, p<0.001), age at first manifestation (β = 0.197, p = 0.017), PANSS negative score (β = −0.414, p<0.001) and PANSS general psychopathology score (β = 0.313, p = 0.025). OXT rs53576 polymorphism, gender, duration of illness and PANSS positive score were not significant as independent predictors. The model was not significant for IRI ‘perspective taking’ or IRI ‘personal distress’ as dependent variables.

To explore possible indirect effects of OXTR rs2254298 polymorphisms on ‘empathic concern’ through partial mediation by PANSS general or negative scores, or age of onset of schizophrenia, mediation analysis [97] was conducted. OXTR rs2254298 significantly predicted PANSS general psychopathology scores (β = −0.193, p = 0.020), but it showed only a trend on PANSS negative symptoms (β = −0.151, p = 0.070) and no effect on age of onset of schizophrenia (β = −0.037, p = 0.663). Comparisons of the direct effects of OXTR rs2254298 on IRI ‘empathic concern’ (β = −0.280, p<0.001) and β-values from simultaneous regression of IRI ‘empathic concern’ on OXTR rs2254298 including each of the 3 potential mediators as additional independents did not indicate mediation effects (with PANSS general psychopathology: β = −0.287, p<0.001; with PANSS negative: β = −0.315, p<0.001; with age of onset: β = −0.273, p<0.001). Moreover, no significant indirect effect could be determined by calculating Sobel’s Z-values in a multiple mediator model (p>0.05) [97].

## Discussion

The potential relationship of allelic variations of OXTR rs2254298 and rs53576 with various psychopathological or temperamental conditions has been investigated in several studies. In this study, we compared basic empathy dimensions and two SNPs within the OXTR gene in schizophrenic patients and healthy controls. Group comparisons of the IRI empathy scores revealed significantly lower self-report ‘perspective taking’ and higher ‘personal distress’ scores in schizophrenia patients compared to controls, replicating our previous finding in a larger independent sample [95]. However, the main result is a significant main effect of OXTR rs2254298 as well as a significant interaction between diagnosis and OXTR rs2254298 on IRI ‘empathic concern’, with schizophrenic patients carrying an AA- or AG-genotype showing highest IRI values.

Our findings might corroborate previous research indicating a possible genetic contribution of OXTR polymorphisms a) to social cognitive functioning and empathy [73], [79], [98] and possibly b) to schizophrenia risk and psychopathology [69]–[72]. Of note, frequencies of both OXTR polymorphisms did not differ between schizophrenic patients and controls in this small sample, thus partially contradicting the findings of [72]. While the negative finding with respect to OXTR rs53576 might be attributed to lower statistical power, OXTR rs2254298 was neither targeted in previous studies of schizophrenic individuals [69], [70], [72] nor identified as associated with the disease in a large Arab-Israeli pedigree study [71]. For this reason, no final statement can be made about a possible contribution of OXTR rs2254298 polymorphisms to schizophrenia vulnerability so far.

Regarding a broader spectrum of disorders of social cognition, preliminary evidence points to an involvement of OXTR rs2254298, whose A-allele was considered a risk for autism spectrum disorder in Chinese Han families [60] and in a Japanese case-control study [63]. Interestingly, studies in European and Israeli samples rather identified the rs2254298 G-allele as the risk variant [62], [84] though three family-based studies did not report significant direct associations of SNP rs2254298 with autism [63], [67], [84]. Of note, IRI results in patients with Asperger syndrome rather suggest difficulties at ‘perspective taking’ and the inference of epistemological mental states and not primarily involve trait interpersonal warmth and sympathy [99]; also for this reason our finding cannot be held indicative of a shared vulnerability between the two disease entities. In contrast, it can be speculated that OXTR polymorphisms might differentially modulate behavioral domains in various disorders in interaction with additional specific pathogenetic factors, genetic variants or medication. This might also explain why the empathy-related phenotype in our study was differentially expressed across genotypes in both experimental groups.

Notably, OXTR rs22454298 showed an association only with the IRI ‘empathic concern’ subscale and not with cognitive empathy (IRI ‘perspective taking’) or self-centered aversive arousal in socio-emotional contexts (IRI ‘personal distress’) that both differed significantly between schizophrenic patients and controls. The impact of OXTR polymorphisms on prosocial attitudes in schizophrenia extends reports of an influence of peripheral OXT levels on prosocial symptom scores [48], emotion recognition [49], [54], [100], social cognition [52] and trust [101] in schizophrenia. In partial contrast to our result, recent evidence from a sample of healthy Chinese individuals indicated an association of OXTR rs2254298 with scores of cognitive empathy, but not with the emotional subscales of the IRI - similar to our study, no associations were detected for rs53576 [79]. However, studies cannot easily be compared due to their different ethnic and cultural backgrounds, and discrepancies between Caucasian and Asian samples as for rs2254298 were reported by several authors [60], [62], [80]. Investigating OXTR SNP rs53576, Rodrigues et al. [73] used a composite measure of all other-oriented, cognitive and emotional IRI scales and observed significantly higher values in healthy GG-carriers. Also Krueger et al. [78] reported higher IRI dispositional empathy and interpersonal trust in 108 healthy men carrying rs53576(GG). Using the same questionnaire, our result substantiates a possible influence of OXTR rs2254298(A>GG), but not OXTR rs53576, on emotional empathy also in schizophrenia.

Although evidence is still conflicting with regard to directionality [80]–[82], the association of OXTR rs2254298(A>GG) with ‘empathic concern’ is consistent with studies of other patient populations indicating respective links with measures of emotional vulnerability [102], [103]. Also, the OXTR rs2254298 GG-genotype seemed to be protective with respect to depressive and anxious symptoms in adolescent girls whose mothers had suffered from depression [104]. In contrast, Feldman et al. [81] reported that parents homozygous for the rs2254298 GG-genotype had lower plasma OXT compared with A allele carriers, and the frequency of parental touch correlated positively with plasma OXT. Recent genetic imaging studies reported an association of OXTR rs2254298(A) with larger bilateral amygdala volumes in healthy Japanese [85] and healthy female adolescents [86]. Tost et al. [87] detected a significant decrease in hypothalamus - but not amygdala - gray matter by voxel-based morphometry, a deficient deactivation of the dACG during emotion processing in Caucasian rs2254298 A-carriers, and a relative decoupling of hypothalamus functional connectivity from dACG and amygdala in male A-allele carriers. While ethnical and methodological aspects of these findings are still discussed [105], results do not contradict a putative link between OXTR rs2254298(A) and a relatively higher emotional reactivity as seen in our study. In schizophrenia, oxytocinergic input may interact with structural [106], [107] and functional abnormalities of the amygdala [108] and its interconnections with dopaminergic structures and prefrontal cortex - and thus contribute to aberrant emotional salience attribution and alterations of social reward circuitry. Variations in the OXT system may therefore partly explain psychotic core symptoms together with specific socio-emotional deficits in schizophrenia [44], [69].

The fact that OXTR rs2254298 specifically impacted other-oriented feelings, but not self-oriented distress, does at first glance not comply with evidence regarding the mitigating role of OXT in emotion regulation per se through its effect on HPA activation [14]. However, it can be hypothesized that the ability to form social bonds and to interpersonally exchange support represents a focal point for OXT-mediated stress regulation. For instance, though previous research confirmed lower stress reactivity in healthy OXTR rs53576(GG) individuals compared to A-allele carriers [73], other studies administrating OXT during stressful experimental situations suggested an interaction between OXT-mediated stress reduction and the presence of social support [38]. This effect was found to be more prominent in individuals with the rs53576(GG) genotype [74]. Moreover, OXT administration in females in a crucial interpersonal situation - namely during the exposure to infant laughter and crying - increased functional connectivity between the amygdala and neural networks subserving emotion regulation, thus probably reducing negative emotional arousal and aversion [109], [110].

Schizophrenic patients carrying an A-allele of OXTR rs2254298 showed significantly higher scores of PANSS general psychopathology, but not of positive or negative symptoms. Although the group of A-carriers comprised comparably more males, patients showed higher scores of ‘empathic concern’. Linear regression analysis identified not gender, but OXTR rs2254298(A), late age of onset of schizophrenia, low PANSS negative, and high PANSS general psychopathology scores as predictors of high self-rated ‘empathic concern’. OXTR rs2254298(A) significantly predicted PANSS general scores. Although mediation analysis in our sample did not confirm significant indirect effects, OXTR rs2254298 A-carriers might represent the more “affective” pole of our schizophrenia sample, with PANSS general scores reflecting the predominance of affective, anxious and psychomotor symptoms. This explanation might be in accord with reports of OXTR rs2254298 impacting the risk for affective disorders [102]–[104]. On the other hand, no significant impact of OXTR polymorphisms was detectable on schizophrenic core symptoms measured with the PANSS positive and negative subscales. While this also could be attributed to insufficient statistical power, results stand in partial contrast to the positive accounts of a therapeutic OXT administration on schizophrenic core - in particular, positive - symptoms [51], [52], [111]. Our results might support the view that the oxytocinergic system exerts its effects on schizophrenic psychopathology by impacting lower-level dispositions, such as anxiety, social motivation and perceptual selectivity [112], [113] and not by a selective influence on social cognition and related core symptoms like delusions or ideas of reference [114]. Moreover, future research might elucidate the functional interplay of OXTR polymorphisms and the short-term regulation of peripheral and central OXT levels during socio-emotional processing and its importance for social dysfunction [115].

Several limitations of the study should be noted. OXTR SNPs were selected on the basis of previous publications. As their functional significance including the existence of influential loci in linkage disequilibrium, as well as regulation and physiology of the cerebral OXT receptor are still not known, genetic associations must not suggest causality. As large effects of genetic variations cannot be expected in complex phenotypes, the limited number of SNPs restricts the validity of our result, while important confounding mechanisms like epistatic factors, polymorphisms of other candidate genes, epigenetic regulation and gene-environment interactions [77], [98], [116] had to be ignored. Low case numbers prevented a detailed comparison of male and female subsamples, although earlier research indicates pronounced sexual dimorphisms in the OXT system [12], [48], [88], [103], [117] and its genetic variations [71], [72], [118]. Also, peripheral OXT levels were not measured, which could have strengthened our result, as preliminary evidence points to a complex relationship of genetic markers, OXT plasma and CSF levels as well as socio-emotional behaviors [17], [81].

Also the categorical approach to schizophrenia does not give consideration to this highly heterogeneous disease entity [119]. Ratings of empathy, psychopathology and also peripheral or central OXT levels might be confounded by medication [50], [56], [57], and a dysfunctional interplay of OXT with neurotransmitters like dopamine [44], [46] or serotonin [83] may disturb feedback regulation between OXT secretion and social context [101] and prevent the detection of subtle effects of OXTR genetic polymorphisms on behavioral measures.

Finally, we used a single self-rating instrument, although of proven validity in schizophrenia [11], to assess empathic dimensions. Our results therefore have to be regarded with caution because of their preliminary nature. In conclusion, we give tentative evidence on the involvement of OXTR genetic variants in empathic functioning in schizophrenia. With respect to the high clinical relevance of social cognition in this disorder [2] and the possible role of OXT as a new pharmacological agent [111] we suggest that an independent substantiation of our results is warranted.

## Supporting Information

Table S1 **MANCOVA of 3 IRI scores in schizophrenia patients (SZ, n = 145) and healthy controls (HC, n = 145); factors: OXTR rs2254298 (GG vs. A carriers), gender and diagnosis, covariate: verbal IQ and educational years.**
(DOCX)
