# Supplementary material for: Association between Oxytocin Receptor Gene Polymorphisms and Self-Rated ‘Empathic Concern’ in Schizophrenia
Source: PLoS One. 2012 Dec 20;7(12):e51882. doi: 10.1371/journal.pone.0051882 (PMC3527477; doi:10.1371/journal.pone.0051882)
Supplement: Table S1 — MANCOVA of 3 IRI scores in schizophrenia patients (SZ, n = 145) and healthy controls (HC, n = 145); factors: OXTR rs2254298 (GG vs. A carriers), gender and diagnosis, covariate: verbal IQ and educational years. (DOCX) [file pone.0051882.s001.docx]

**TABLE S1:** MANCOVA of 3 IRI scores in schizophrenia patients (SZ, n=145) and healthy controls (HC, n=145); factors: OXTR rs2254298 (GG vs. A carriers), gender and diagnosis, covariate: verbal IQ and educational years.

|  | **OXTR s2254298** | **Diagnosis** | **Gender** | **OXTR s2254298 x Diagnosis** | **OXTR s2254298**  **x**  **Gender** | **Diagnosis**  **x**  **Gender** | **Verbal IQ** | **Educ. years** |
| --- | --- | --- | --- | --- | --- | --- | --- | --- |
| **MANCOVA F** [3,279] (Effect size) | **4.720**** (_p_η^2^=0.048) | **18.899*****  (_p_η^2^=0.169) | **5.862*****  (_p_η^2^=0.059) | **4.676**** (_p_η^2^=0.048) | 1.822  (_p_η^2^=0.019) | 0.402 (_p_η^2^=0.004) | **2.797*** (_p_η^2^=0.029) | 1.228  (_p_η^2^=0.013) |
| Post hoc ANOVA F [1,281] | | | | | | | | |
| IRI 'perspective taking' (R^2^_adj_=0.061) | 3.270 | 2.819 | 0.878 | 0.225 | 1.159 | 0.012 | **7.861**** | 1.176 |
| IRI 'empathic concern' (R^2^_adj_==0.070) | **14.114***** | 2.022 | **9.456**** | **7.164**** | 1.118 | 0.799 | 2.862 | 0.420 |
| IRI 'personal distress' (R^2^_adj_==0.201) | 0.009 | **50.012***** | **8.170**** | **4.382*** | 3.780 | 0.158 | 0.122 | 1.256 |
| Significant results are indicated in bold type (*p<0.05, **p<0.01, ***p<0.001). | | | | | | | | |
